# The Global Fund, Governance, and Accountability

**DOI:** 10.5334/aogh.2530

**Published:** 2019-05-20

**Authors:** Eric Goosby

**Affiliations:** 1 Center for Implementation Sciences, University of California, San Francisco, US; 2 United Nations Special Envoy on Tuberculosis, US

The Global Fund began in 2002 as a unique experiment to fight the massive global health diseases of AIDS, tuberculosis, and malaria, which combined counted for millions of deaths each year. Its innovative financing mechanism allowed it to prioritize local planning of unmet needs and allocate appropriate resources. The Global Fund’s financing mechanism was created to address the inherent governing challenges presented when distributing aid, such as not having all of the relevant players at the table when decisions were being made. Now in its seventeenth year, it is fair to say that this unique funding mechanism is not only tackling the three diseases, it is creating an environment that brings about positive changes in governance areas such as transparency and accountability and has the significant potential to tackle issues like fighting corruption. Simply put, investment in the Global Fund is now an investment that goes beyond the critical work of just saving lives.

In their paper, ‘Governance and Health Aid from the Global Fund: Effects Beyond Fighting Disease,’ Matthew Kavanagh and Lixue Chen make the argument that there is emerging evidence to support this thesis. It begins to look at whether practices and institutions like the decision-making of Country Coordinating Mechanisms (CCMs), as they work to demonstrate an impact on populations based on outcomes or validated surrogate markers, may contribute toward improved governance on a broader level. The paper opens the door to showing both the importance of the Global Fund to program expansion and quality controls. It also adds information for Ministry of Health-Policy Leaders and all country-specific stakeholders that they must ultimately develop and implement local systems that assume responsibility for management oversight, monitoring, and evaluation as domestic dollars move to dominate disease spending.

To accomplish this, the Global Fund has set up a governance process that allows for the convening of stakeholders involved in planning and implementing both domestic and donor resources. This process also serves as the body that defines unmet needs and makes allocation decisions in multiple fiscal years, allowing for real time reforms in the next fiscal year, and defines a body that can be held accountable by those who use and need the services.

Non-governmental organizations (NGOs), civil society, multilateral, and bilateral organizations are all involved in the Global Fund model. CCMs address transparency, participation and representation at the country and the global levels and define and correct conflict of interests amongst the stakeholders. Audits and local funding agents ensure checks and balances in financial resources. Donor resources have a tendency to create a parallel system of reporting and auditing. When budget control is vested in Geneva it dilutes the ability of those using the services to hold management accountable. Moreover, lack of country autonomy in use of funds undermines local ownership of data analysis and quality improvement. It precludes the ability of the in-country policy makers to link allocation decisions to outcomes at the population level. By contrast, the Global Fund approach strengthens local decision making, enabling the CCM to integrate multiple resource streams and, in the same fiscal year, identify duplication, redundancy, and both effective and ineffective programing to improve population outcomes in real time.

The infrastructure created by the CCM with the convening of the principal stakeholders in government, implementing health system professionals, civil society, and donors becomes the appropriate site to plan and implement additional programs needed to expand to the service needs of non-communicable diseases and move to a more sustainable universal health care coverage.

As one examines the ability of the Global Fund to address other issues that naturally stem from diagnosis and treatment of diseases, it is critical to remember that it is not only a unique funding mechanism, it has the flexibility to change if change is for the greater good. When I was the US Global AIDS Coordinator during the Obama Administration, I also sat on the Global Fund Board. We took a serious look at the structure and workings of the Global Fund. This effort resulted in several actions that are reaping dividends today: 1) strengthened oversight of the use of funds where donors hold the Global Fund accountable and the Global Fund holds countries accountable; 2) transformation of the funding model from giving countries grants to a stakeholder governance model that sets goals with stakeholders prioritizing what is actually needed on the ground, giving CCMs more decision-making authority; and 3) above all, ensuring the Global Fund enforces stricter mechanisms to strengthen transparency and accountability. As the paper states, “these levels (of transparency and accountability) are unusually high among aid programs.” Evidence that this is having the intended effect is good news indeed.

The Global Fund has saved 27 million lives since its inception. It can save countless more with a continued investment in its work. Moreover, it can spark greater government accountability and regulatory quality. That’s aid in action.
